# Adult Age Differences in the Use of Conceptual Combination as an Associative Encoding Strategy

**DOI:** 10.3389/fnhum.2019.00339

**Published:** 2019-10-10

**Authors:** Heather D. Lucas, Resh S. Gupta, Ryan J. Hubbard, Kara D. Federmeier

**Affiliations:** ^1^Department of Psychology, Louisiana State University, Baton Rouge, LA, United States; ^2^Vanderbilt Brain Institute, Vanderbilt University, Nashville, TN, United States; ^3^Department of Psychology and Beckman Institute, University of Illinois Urbana-Champaign, Urbana, IL, United States

**Keywords:** conceptual combination, associative memory, cognitive aging, ERPs, imagery

## Abstract

It is well-established that aging impairs memory for associations more than it does memory for single items. Aging also impacts processes involved in online language comprehension, including the ability to form integrated, message-level representations. These changes in comprehension processes could impact older adults’ associative memory performance, perhaps by reducing or altering the effectiveness of encoding strategies that encourage semantic integration. The present study examined age differences in the use of a strategy termed conceptual combination, which involves integrating two words (e.g., “winter” and “salad”) into a single concept (“a salad for winter”). We recorded ERPs while participants studied unrelated noun pairs using a strategy that either did or did not encourage conceptual combination. We also varied the concreteness of the first noun in each pair in order to measure compositional concreteness effects, or ERP differences at the second noun due to the concreteness of the first noun. At the first nouns, older adults showed word-level concreteness effects that were similar to those of younger adults. However, compositional concreteness effects were diminished in older adults, consistent with reduced semantic integration. Older adults’ associative memory performance was better for word pairs studied during the conceptual combination task versus the non-combinatory encoding task; however, the magnitude of the age-related associative memory deficit did not differ between tasks. Finally, analyses of both memory accuracy and trial-by-trial ratings of perceived combination success suggested that older adults had disproportionate difficulty applying the conceptual combination strategy to word pairs that began with abstract nouns. Overall, these results indicate that changes to integrative language processing that occur with age are not independent of – and may sometimes exacerbate – age-related memory decline.

## Introduction

A hallmark of the typical aging process is a reduced ability to learn and remember new information. However, aging does not uniformly affect all types of memory. Memory for associative or relational information (e.g., arbitrary pairings or groupings of stimuli, such as face-name pairings) is particularly susceptible to age-related decline, whereas the ability to remember single items is relatively spared (for a recent meta-analysis, see Old and Naveh-Benjamin, 2008). These behavioral findings converge well with data from studies examining age effects on brain structure and function. Associative memory tasks place strong demands on the hippocampus and certain regions of the prefrontal cortex (Vargha-Khadem et al., 1997; Preston and Eichenbaum, 2013; Addis et al., 2014), both of which decrease in volume and integrity (Raz et al., 2005) as well as encoding-related activity (Dennis et al., 2008) across the adult lifespan. By contrast, item memory has been linked to the surrounding medial temporal lobe (MTL) cortex, particularly the perirhinal cortex (e.g., Davachi, 2006), which is less susceptible to changes with age (Dickerson et al., 2009).

In recent years, there has been a growing interest in the idea that individuals from populations with reduced hippocampal integrity may be able to develop strategies to increase their ability to rely on spared item memory to remember certain types of associations. Indeed, under the right circumstances, otherwise arbitrary associations can be represented in memory in a manner that is relatively *unitized* or item-like. For example, in a neuroimaging investigation of color-object associations (Diana et al., 2010), the perirhinal cortex was found to be more active during retrieval when the color was initially encoded as intrinsic to, rather that arbitrarily co-occurring with, the object. Other studies (e.g., Quamme et al., 2007; Haskins et al., 2008) have examined the extent to which a form of processing termed *conceptual combination* can be strategically applied to arbitrary word pairs in order to achieve relatively unitized memory representations. Conceptual combination refers to the processing of noun pairs as modifier-head dyads that together form new, emergent concepts. For example, applying conceptual combination to the word pair “dog spoon” might prompt an interpretation such as “a spoon that was designed specifically to feed dogs.”

The link between conceptual combination and unitization has been established in studies of patients with amnesia due to damage to the MTL. Quamme et al. (2007) asked both healthy participants and patients with MTL lesions to study unrelated word pairs under two conditions that either did or did not promote conceptual combination. In a so-called separate encoding condition, participants were shown each word pair along with a sentence with two corresponding blanks (e.g., “The ____ could be seen from the _____” for the word pair “cloud-lawn”). By contrast, in the compound encoding condition, each word pair was accompanied by an experimenter-generated definition that served to combine the words into a novel but meaningful concept (e.g., “a yard used for sky-gazing”). On a later test of associative recognition, participants with hippocampal damage performed markedly better when tested on items they studied in the conceptual combination condition. However, participants with more widespread temporal lobe injury that also encompassed the perirhinal cortex did not show this advantage, nor did healthy controls for whom both hippocampal and perirhinal processing were presumably intact (see Haskins et al., 2008, for converging evidence from fMRI).

Together, these findings suggest that the process of conceptually combining novel word pairs – when successful – can reduce the associative memory deficit observed in populations characterized by hippocampal decline, potentially including older adults (Ahmad et al., 2014; Bastin et al., 2013). However, very little is known about how the conceptual combination process itself might change with age, particularly when applied in an *ad hoc*, flexible manner to word pairs that do not correspond to pre-existing definitions. Indeed, one recent study (Kamp et al., 2018) produced the counterintuitive finding that older adults’ associative memory for unrelated word pairs was worse when those word pairs were presented as part of an experimenter-defined compound phrase versus as part of a sentence – a pattern that is opposite to what has been found in patients with hippocampal amnesia.

Of note, there is evidence that age can impact processes related to semantic integration, or the construction and maintenance of message-level representations from language. Several studies using event-related potentials (ERPs, reviewed in Wlotko et al., 2010) have demonstrated that older adults show a reduced tendency to use sentential context to guide or constrain the processing of upcoming words. Analyses focusing on the N400, an electrophysiological index of the processing demands associated with semantic access, have been informative in this regard. N400 amplitudes are modulated by item-level lexical attributes, such as word frequency and orthographic neighborhood size, as well as the “fit” of incoming information with the preceding semantic or syntactic context (Kutas and Federmeier, 2011). Compared to younger individuals, older adults demonstrate a decreased sensitivity of N400 potentials to contextual information, combined with a spared or even increased sensitivity to lexical characteristics (Payne and Federmeier, 2018), suggesting a diminished capacity for rapid and flexible construction and/or use of context from semantic information.

It seems plausible that this reduced ability to build up contextual information and/or integrate it with incoming stimulus-based information could contribute to difficulties in implementing strategies that involve conceptual combination, particularly when applied to word pairs that are pre-experimentally unrelated. Of particular relevance, Huang and colleagues (Huang et al., 2010, 2012) demonstrated that age effects on semantic integration extend to simple two-word phrases, not unlike the stimuli used in many associative memory experiments. In these studies, ERPs were recorded while younger and older adults viewed a series of common nouns, each of which was alternately preceded by either a concrete or an abstract adjective (for example, “hilly farm” versus “productive farm”). Both age groups showed robust concreteness effects on ERP responses to the adjectives, consistent with reports that aging does not reduce sensitivity to lexical characteristics. In particular, relative to abstract adjectives, concrete adjectives elicited more negative N400 potentials, as well as enhanced amplitudes of a late frontal negativity, sometimes referred to as the N700, which has been linked to either visual imagery (West and Holcomb, 2000; Welcome et al., 2011; Gullick et al., 2013) or to a modality-independent feature integration process (Barber et al., 2013). Importantly, only in the young adults were concreteness effects evident at the compositional level, or in response to the same head nouns as a function of the concreteness of the preceding adjective. Mirroring world-level concreteness effects, nouns that had been modified by a concrete adjective elicited smaller N400 potentials and larger N700 potentials than did the same nouns when modified by abstract adjectives. By contrast, compositional concreteness effects were absent in the older adults, suggesting a reduced ability to incorporate the features specified by the adjective into the meaning elicited by the head noun.

In the present study, we build on these findings by examining whether age differences are also present during a noun-noun conceptual combination task, similar to tasks that have been suggested to promote the formation of unitized memory representations. In a recent study (Lucas et al., 2017), we demonstrated that the N400 and N700 compositional concreteness effects found for adjective-noun processing were also evident during the processing of unrelated noun pairs (e.g., “road salad” versus “idea salad”). The later of these two compositional concreteness effects (N700) was found to be task specific, in that N700 differences were present on the second noun only when participants were encouraged to engage in conceptual combination by attempting to generate a sensible compound meaning for each word pair. When the same word pairs were processed in a task that involved comparing the relative frequency of the concepts denoted by the two words, only N400 compositional concreteness effects were present. Moreover, a subsequent free recall test revealed that word pairs that had been initially processed via conceptual combination were represented in memory in a more holistic manner, in that they tended to be recalled from memory as pairs rather than individual items, consistent with the notion that conceptual combination promoted unitization.

Together, these data suggest that (1) N700 concreteness effects, when present at the compositional level (e.g., on the same lexical item as a function of the concreteness of a preceding modifier), reflect an aspect of semantic integration that can be deployed in a top-down manner to support the ability to interpret novel concepts, and (2) doing so promotes the formation of strong and perhaps unitized associative memory representations in younger adults. As such, the design employed by Lucas et al. (2017) provides a starting point to examine the extent to which age-related decreases in integrative processes associated with language comprehension can impact the online conceptual combination process, thereby limiting older adults’ use of conceptual combination as a strategy to remediate associative memory deficits.

In this experiment, we used the same materials and procedures from Lucas et al. (2017) in a sample of healthy older adults. ERPs were recorded as participants studied unrelated noun pairs (either abstract-concrete or concrete-concrete) under instructions that either did or did not emphasize conceptual combination. In conceptual combination blocks, participants were asked to generate plausible compound definitions for each of the word pairs and provide trial-by-trial subjective ratings of their success in doing so. In frequency-comparison blocks, participants judged whether the concept denoted by the first word (W1) was one that they encountered more or less often relative to the concept denoted by the second word (W2). Given prior evidence for spared word-level processing in older adults, we predict that, regardless of task, older adults will show the canonical pattern of lexical-level ERP concreteness effects on the W1s, including larger N400 responses and a sustained frontal negativity to concrete as compared with abstract words. However, in line with Huang et al. (2012), we do not expect that older adults will show the N700 compositional concreteness effects that were previously found in younger adults during conceptual combination. Rather, we expect that the N700 ERPs elicited by the W2s in older adults will be insensitive both to task demands (frequency comparison versus conceptual combination) and to the concreteness of the preceding W1. To examine age effects, we also compared older adults’ ERP data to those of the younger adults described in Lucas et al. (2017). We predict that significant age differences will be present in the magnitude of compositional, but not item-level, concreteness effects, and that these differences will be specific to the conceptual combination task.

In addition to examining encoding-related ERPs, we conducted multiple complementary analyses to better understand the underlying mechanisms and downstream effects of flexible conceptual combination in older adults. First, we tested associative recognition memory after each block to examine the extent to which the benefits enjoyed by young adults following conceptual combination are also present in older adults. Second, we examined whether participants’ trial-by-trial ratings of conceptual combination success predicted subsequent memory for word pairs studied during the conceptual combination task. To foreshadow these results, we found that participants from both age groups assigned significantly higher ratings to word pairs that they went on to remember versus those that they did not. This finding provides behavioral evidence for overlap between the online processes involved in flexible conceptual combination and those that facilitate associative memory formation. As such, we build on these results by using a single-trial analysis approach to assess relationships between ERPs linked to conceptual combination and perceived success across trials.

## Materials and Methods

### Participants

Twenty-four older adults (13 female, mean age = 68.6 years, range = 61–79 years) from the Champaign–Urbana area participated in the study and were compensated $10/hour. All were right-handed and native speakers of English. An additional five individuals completed the experiment but were excluded from analyses due to difficulty following task instructions (*n* = 1), poor EEG data quality (*n* = 2), or because they scored lower than a 51/57 on a modified version of the Mini Mental State Examination (*n* = 2), which was administered prior to beginning the study. The average score for the included participants was 54.5 (range 51–57).

To examine age effects, key variables of interest were compared with the sample of 24 younger adults reported in Experiment 1 of Lucas et al. (2017). These participants were from the University of Illinois and surrounding areas. All were right-handed and native speakers of English. The mean age of this sample was 21 years (range = 18–24 years, 18 female). The study design, equipment, and procedures employed in the young adult experiment were identical to those in the present experiment, except that the Mini Mental State Examination was not administered to the younger adults.

### Stimuli

The stimuli were the same as those used in Lucas et al. (2017) and consisted of 144 noun pairs (72 abstract-concrete pairs and 72 concrete-concrete pairs) formed from a set of 72 abstract nouns and 216 concrete nouns. Abstract and concrete nouns had a mean concreteness rating of 280 (range = 232–349) and 574 (range = 500–646), respectively, a mean imageability rating of 383 (range = 262–551) and 564 (range = 424–667), respectively, and a mean Kucera-Francis written frequency rating of 56 (range = 1–447) and 41 (range = 1–442), respectively, according to the Medical Research Council database described by Coltheart (2007, http://websites.psychology.uwa.edu.au/school/MRCDatabase/uwa_mrc.htm).

To construct the word pairs, each of the 72 abstract nouns was paired with a concrete noun of comparable frequency, familiarity, and length. These 144 nouns served as the first words (W1s). Each pair of yoked W1s was assigned two randomly chosen words from the remaining set of 144 concrete nouns to serve as second words (W2s). By creating two sets of word pairings in this manner, we were able to counterbalance the frequency with which each W2 was preceded by a concrete versus an abstract W1 across participants. The frequency with which each word pair was presented in a conceptual combination block versus a frequency-comparison block was also counterbalanced.

We manually inspected and adjusted noun pairings to eliminate pairs with clear pre-experimental meanings (e.g., “flower store”). In addition, we used the University of South Florida Free Association Norms database (Nelson et al., 2004) to obtain free association data for 109 of the 144 W1s. For these words, we were able to confirm that the corresponding W2s were not forward associates of the W1s.

### Procedures

The procedures were the same as those of Lucas et al. (2017). Each participant completed four study-test blocks, two of which were conceptual combination blocks and two of which were frequency-comparison blocks. Both blocks of each type were completed consecutively, and presentation order was counterbalanced across participants.

#### Study Phases

**FIGURE 1:**
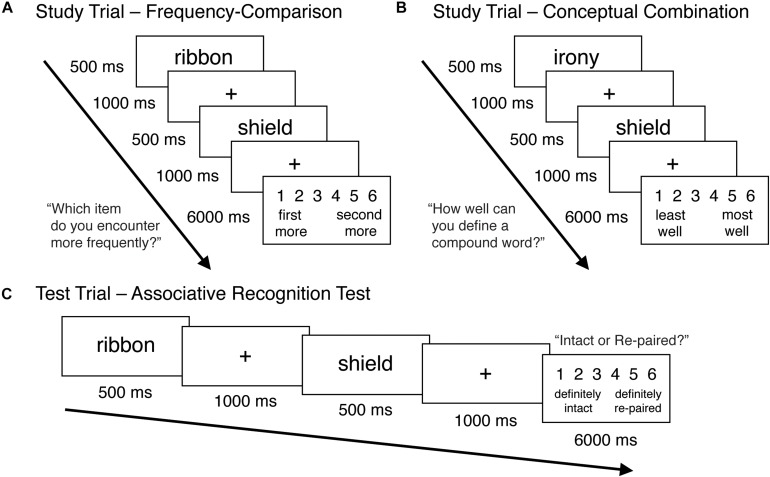
Sample study trial for the frequency-comparison task **(A)** and conceptual combination task **(B)**, as well as a sample intact trial from the associative recognition test **(C)**. Adapted from Figure 1 of Lucas et al. (2017).

Sample study trials for the frequency-comparison and conceptual combination tasks are depicted in Figures 1A,B, respectively. Each study phase consisted of 36 word pairs (18 abstract-concrete and 18 concrete-concrete), which were presented in a random order, as well as one primacy and one recency buffer trial. In each study trial, a W1 was presented for 500 ms, followed by a fixation cross presented for 1000 ms, and then a 500 ms presentation of the W2. A fixation cross then appeared again for 1000 ms, followed by a 5000 ms prompt in which the appropriate rating scale was displayed, and participants were asked to make a response.

During the conceptual combination blocks, participants were asked to use a 1–6 scale to indicate the relative ease with which they could generate a definition for the compound phrase formed from the two words. For half of the participants, a rating of “1” was assigned to pairs for which no meaning came to mind at all, and a rating of “6” corresponded to word pairs that were easiest to clearly define. Buttons 2–5 reflected intermediate levels of difficulty. These mappings were reversed for the remaining participants. To help ensure that the ratings would provide information about the relative ease of defining each word pair, we asked participants to make these ratings on a relative basis and to use the six buttons approximately evenly. We also emphasized that, while an attempt should be made on each trial to construct a meaningful definition, it was likely that for some trials no definition might come to mind, and that participants should press Button 1 (or 6, depending on counterbalancing) if they were unable to generate any definition.

During the frequency-comparison blocks, participants were instructed to use a 1–6 scale to indicate which of the items denoted by the two words is encountered more frequently. For half of the participants, a rating of “1” indicated a much greater frequency of the first word, while a rating of “6” indicated a much greater frequency of the second word. Intermediate buttons were used for less extreme frequency differences. Ratings were reversed for the remaining participants.

#### Test Phases

In between the study and test phase, participants completed a brief (30 s) distractor task in which they were asked to count backwards in twos from a randomly generated number between 300 and 600. After the distractor task, an associative recognition test was administered. A sample test trial is depicted in Figure 1C. Each test phase consisted of the 36 pairs from the most recent study block, half of which were intact (presented in the same pairing as they had been during the study phase), and half of which were re-paired (paired with a different word from the same block). Re-pairings were determined randomly for each participant within each counterbalancing condition, with the following constraints: (1) W1 words remained first in each pair and were always re-paired with W2 words, (2) each W2 was paired with a W1 at test with the same concreteness status as its W1 from the study phase, and (3) an equal number of abstract-concrete and concrete-concrete word pairs were presented as intact or repaired within each block. Test blocks began with the primacy and recency buffers of the previous study block, which were used as practice buffer trials and not included in analyses.

The timing and structure of the test trials were the same as the study trials, except that participants were instructed to provide confidence ratings as to whether each word pair was intact or re-paired on a scale of 1–6. For half of the participants, Button 1 corresponded to a high degree of confidence that a pair was intact, while Button 6 indicated a high degree of confidence that the pair was re-paired. Buttons 2 and 5 were used to indicate medium levels of confidence, and buttons 3 and 4 denoted low levels of confidence. These ratings were reversed for the remaining participants.

Before beginning the experiment, participants completed brief, six-trial practice blocks for each of the two study tasks, followed by a practice associative recognition test. During the practice conceptual combination block, participants were asked to verbally describe the definition they produced for each trial and explain their choice of button press. After the participant described their definition (or expressed an inability to come up with a definition), the experimenter offered multiple different examples of possible responses to reinforce the instructions.

### Electrophysiology

ERPs were extracted from scalp electroencephalographic recordings from 26 Ag/AgCl electrodes spaced evenly over the head. Voltages were referenced online to a left mastoid electrode and re-referenced offline to averaged left and right mastoids. Electrode impedances were kept below 5 kΩ. Signals were recorded with a bandpass filter of 0.01–100 Hz and sampled at a rate of 1000 Hz (BrainVision system). A bandpass filter of 0.1–30 Hz was applied offline prior to statistical analyses. An additional 10 Hz low-pass filter was applied to grand averages for display purposes only. Data preprocessing and analyses were conducted using EEGLAB (Delorme and Makeig, 2004).

Eye movements and blinks were recorded from three additional electrodes below the center of the left eye and on the outer canthus of each eye. Datasets in which more than 25% of epochs contained blink artifacts (*n* = 16) were individually subjected to independent components analyses using Adaptive Mixture ICA (AMICA, Palmer et al., 2012), after which blink components were identified using a combination of manual inspection and by calculating the extent to which activity in each component correlated across time with activity in the bipolar eyeblink channel (calculated as the difference between the VEOG channel and channel LMPF, which is located at the front of the head above the left eye). Blink components were then removed from the EEG only on those trials that were identified as containing blink artifacts. For datasets in which <25% of trials contained blink artifacts (*n* = 8), blink trials were excluded from analyses and no ICA was performed. We then screened for trials containing artifacts due to saccades, muscle activity, and residual eyeblink activity using a simple rejection threshold of ± 75 μV on any scalp channel and a moving window rejection threshold ±40 μV (based on 200 ms windows and a window step of 10 ms) in the bipolar eyeblink and bipolar horizontal eye movement channels. These rejection decisions were then titrated individually using condition-blinded visual inspection to maximize correct rejection of artifacts and minimize loss of clean data. All told, an average of 8.3% of trials (range = 0.08–22.2) were excluded from analysis for each participant.

**FIGURE 2:**
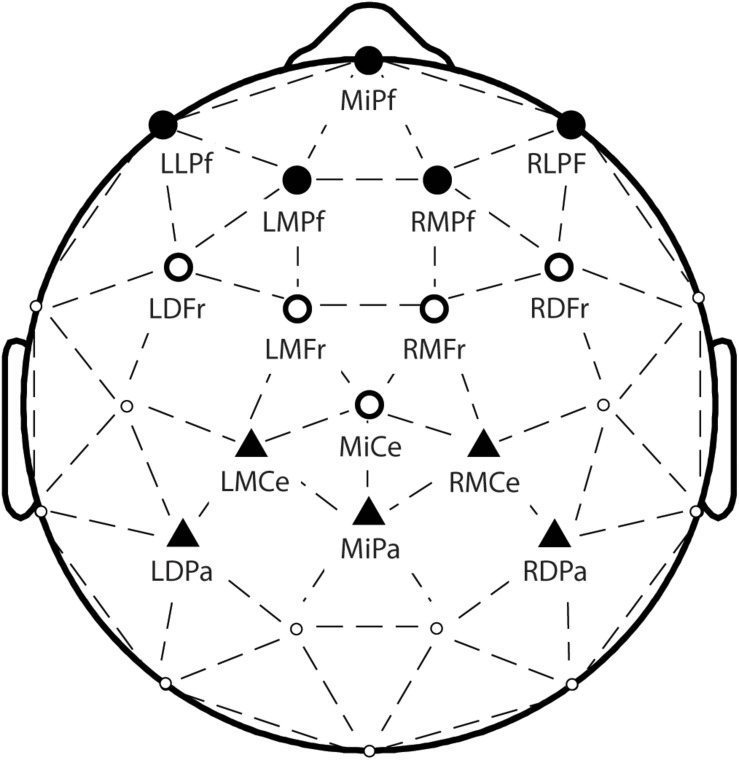
Topographical plot of electrodes, with labels provided for the electrodes included in analyses. Black circles, white circles, and black triangles correspond to the anterior frontal, frontocentral, and parietal clusters, respectively.

Each trial consisted of a 1000 ms epoch time-locked to stimulus onset. The mean amplitude of a 200 ms window prior to stimulus onset was subtracted to correct for baseline variability. As in Lucas et al. (2017), statistical comparisons were performed on amplitudes averaged over an anterior frontal electrode cluster (channels MiPf, LLPf, RLPf, LMPf, and RMPf), a frontocentral electrode cluster (channels LDFr, RDFr, LMFr, RMFr, and MiCe) and a parietal electrode cluster (MiPa, LMCe, RMCe, LDPa, RDPa). These channel locations are depicted in Figure 2. The first letter(s) of the abbreviations denote left (L), right (R), and midline electrodes (Mi). The second letter describes lateral (L), medial (M), and dorsal (D) locations. The final letters denote anteriority, as prefrontal (Pf), frontal (Fr), central (Ce), and parietal (Pa). Occipital and temporal electrodes were not analyzed, as no experimental effects were expected to occur in these regions.

ERP comparisons were performed using repeated-measures ANOVAs (criterion *p* = 0.05) over the windows of 300–500 ms (to capture N400 effects) and 700–1000 ms (to capture N700 effects), consistent with Lucas et al. (2017). The window of 300–500 ms was chosen for N400 analyses based on extensive prior research (e.g., Federmeier and Laszlo, 2009; Kutas and Federmeier, 2011). N700 latencies in previous studies have varied somewhat but tend to occur in the proximity of 700 ms (Holcomb et al., 1999; West and Holcomb, 2000; Huang et al., 2010; Welcome et al., 2011; Barber et al., 2013; Gullick et al., 2013). Mauchly’s Test for Sphericity was used to test for sphericity violations when analyses involved three or more levels of a repeated measure, and the Greenhouse-Geisser correction was applied whenever non-sphericity was present.

We used a twofold strategy to compare ERP effects across age groups. Our first approach was to compute difference waves for each age group via a point-by-point subtraction of the ERP responses for the relevant contrast (e.g., concrete – abstract words) over the 300–500 ms and 700–1000 ms time windows, and then enter the results into a one-way, between-subjects ANOVA with age group as a factor. This analysis method is functionally the same as testing the age × condition interaction.

Our second approach to examining age differences was to submit older and younger adults’ difference waves to independent samples, two-tailed permutation tests based on the cluster mass statistic (Bullmore et al., 1999) using a family-wise alpha level of 0.05. This approach has the advantage of being data-driven and less subject to bias or constraint by *a priori* selections of analysis windows and electrode sites while still maintaining statistical power to detect differences, although power is still lower relative to traditional mean-amplitude based analyses (Groppe et al., 2011). ERPs were first down-sampled to 100 Hz, creating 10 ms time bins. Permutation tests included all time bins between 300 and 1000 ms and all fifteen anterior frontal, frontocentral, and posterior electrodes. Analyses were conducted using the clustGRP function of the Mass Univariate ERP Toolbox (Groppe et al., 2011), which identifies spatiotemporal clusters that differ between conditions by conducting independent samples *t*-tests at each electrode and time bin using the original data and 2500 random between-subject permutations. Neighboring t-scores with uncorrected *p*-values of 0.05 or less are grouped into clusters, and all of the t-scores within a given cluster are summed together to calculate the cluster mass. Finally, a *p*-value is assigned to each cluster by comparing the cluster masses of the observed data with an estimate of the null distribution based on the maximal cluster masses of the random permutations. Electrodes within 5.44 cm of each other were considered spatial neighbors, and adjacent time points were considered temporal neighbors.

## Results: Behavior

### Study-Phase Ratings

Table 1A shows the average proportion of abstract-concrete and concrete-concrete trials assigned each rating on the six-point ease-of-definition scale during the conceptual combination encoding blocks. A paired *t*-test revealed that the average rating assigned to concrete-concrete trials (mean = 4.12, se = 0.12) was significantly higher than that assigned to abstract-concrete trials [mean = 3.86, se = 0.14, *t*(23) = 3.81, *p* < 0.001, Cohen’s *d* = 0.78].

**TABLE 1:** Mean proportion of abstract-concrete (abs-con) and concrete-concrete (con-con) trials assigned each rating on: (A) the six-point ease-of-definition scale in the conceptual combination task, or (B) the six-point relative frequency judgment in the frequency-comparison task.

**(A)**		**Harder to define**	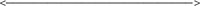		**Easier to define**	
	
	**“One”**	**“Two”**	**“Three”**	**“Four”**	**“Five”**	**“Six”**

Abs-Con	0.15 (0.02)	0.17 (0.02)	0.11 (0.02)	0.08 (0.02)	0.20 (0.03)	0.28 (0.04)
Con-Con	0.13 (0.02)	0.15 (0.02)	0.09 (0.02)	0.10 (0.02)	0.21 (0.02)	0.33 (0.04)

**(B)**		**First word more frequent**	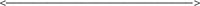		**Second word more frequent**	
	
	**“One”**	**“Two”**	**“Three”**	**“Four”**	**“Five”**	**“Six”**
Abs-Con	0.20 (0.03)	0.10 (0.02)	0.12 (0.02)	0.14 (0.03)	0.14 (0.02)	0.30 (0.04)
Con-Con	0.27 (0.03)	0.11 (0.02)	0.11 (0.02)	0.11 (0.02)	0.12 (0.02)	0.28 (0.03)

Table 1B shows the average proportion of abstract-concrete and concrete-concrete trials assigned each rating on the six-point frequency-comparison scale during the frequency-comparison encoding blocks. Average ratings for concrete-concrete word pairs (mean = 3.52, se = 0.06) were significantly lower than were ratings assigned to abstract-concrete words pairs [mean = 3.81, se = 0.08, *t*(23) = 3.98, *p* < 0.001, Cohen’s *d* = 0.81]. Thus, participants reported encountering the concepts denoted by the abstract W1s less frequently (when compared to the W2s) relative to the concepts denoted by the concrete W1s.

### Associative Recognition Performance

**FIGURE 3:**
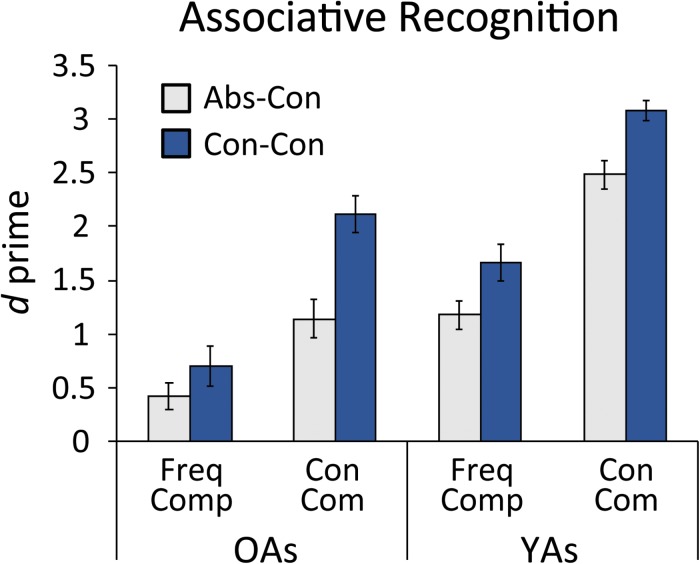
Associative recognition performance as indicated by discrimination sensitivity (*d* prime), subdivided by task and word pair type. YAs, younger adults; OAs, older adults; Freq Comp, frequency comparison task; Con Com, conceptual combination task; Abs-Con, abstract-concrete word pairs; Con-Con, concrete-concrete word pairs. The younger adult data from Lucas et al. (2017) are included for comparison. Although the younger adults outperformed the older adults in all four conditions, the greatest age difference was found for abstract-concrete words in the conceptual combination task. Error bars denote standard error of the mean.

To assess associative recognition memory, discrimination sensitivity (*d’*) was calculated separately for each combination of task and W1 concreteness level. *D’* measures the accuracy with which participants discriminate between studied and unstudied items, and is obtained by subtracting the z-transform of the “false alarm” rate (the proportion of re-paired items incorrectly endorsed as “intact”) from the z-transform of the “hit” rate (the proportion of intact items correctly endorsed as “intact”). The results are depicted in Figure 3. To formally assess the effects of the task and concreteness manipulations on associative memory, a 2 (task: compare/combine) × 2 (W1 type: abstract/concrete) repeated-measures ANOVA was performed on *d’* values. A significant main effect of task emerged [*F*(1, 23) = 50.64, *p* < 0.001, η_p_^2^ = 0.68], indicating higher recognition accuracy in conceptual combination blocks relative to frequency-comparison blocks. In addition, a main effect of W1 type [*F*(1, 23) = 37.14, *p* < 0.001, η_p_^2^ = 0.62] revealed greater accuracy for concrete-concrete relative to abstract-concrete word pairs. The task × W1 type interaction was also significant [*F*(1, 23) = 10.58, *p* = 0.004, η_p_^2^ = 0.32]. Follow-up paired *t*-tests indicated that the beneficial effect of W1 concreteness was significant in the conceptual combination condition [*t*(23) = 6.35, *p* <0.001, Cohen’s *d* = 1.28], but only marginally significant in the frequency-comparison condition [*t*(23) = 2.00, *p* = 0.06, Cohen’s *d* = 0.41].

Unsurprisingly, older adults’ recognition memory was lower than that of young adults in Lucas et al. (2017), for whom d’ values were 1.18 and 1.66 for abstract-concrete and concrete-concrete word pairs in the frequency-comparison condition, and 2.48 and 3.08 for abstract-concrete and concrete-concrete word pairs in the conceptual combination condition, respectively. Indeed, combining these datasets by re-running the ANOVA with age included as a between-subjects factor produced a significant main effect [*F*(1, 46) = 40.17, *p* < 0.001, η_p_^2^ = 0.47]. This comparison also revealed a significant 3-way interaction between age, task, and W1 concreteness [*F*(1, 46) = 4.78, *p* = 0.03, η_p_^2^ = 0.09], suggesting that age differences in the beneficial effects of the conceptual combination over the frequency-comparison task differed across levels of W1 concreteness. Follow-up tests revealed that age differences in associative recognition for concrete-concrete word pairs were equivalent across the two study tasks, as indicated by a non-significant age × task interaction [*F*(1, 46) = 0.00, *p* = 0.98, η_p_^2^ = 0.00]. By contrast, this interaction was significant for abstract-concrete words, [*F*(1, 46) = 5.21, *p* = 0.03, η_p_^2^ = 0.10], reflecting the fact that age differences were *larger* in the conceptual combination relative to the frequency task.

Finally, a series of independent-samples *t*-tests revealed that younger adults outperformed their older counterparts in all four task-W1 combinations [*p*s < 0.001]. However, the effect size of this age difference was larger for abstract-concrete words in the conceptual combination task (Cohen’s *d* = 1.76) compared to the other three conditions (1.46, 1.25, and 1.09 for concrete-concrete word pairs in the conceptual combination task, abstract-concrete pairs in the frequency-comparison task, and concrete-concrete-pairs in the frequency-comparison task, respectively).

### Study-Phase Ratings and Associative Memory

An additional analysis assessed the relationship of participants’ ease-of-definition ratings during the conceptual combination task to subsequent recognition accuracy. A one-way within-subjects ANOVA indicated that, as with the younger adults in Lucas et al. (2017), the average rating given at study by the older adults was significantly greater for items that were later remembered (mean rating = 4.24, se = 0.14) relative to those that were later forgotten [mean rating = 3.11, se = 0.15, *F*(1, 23) = 60.27, p < 0.001, η_p_^2^ = 0.72]. For comparison, the younger adults assigned mean ratings of 4.06 and 2.88 to later-remembered and later-forgotten items, respectively. Re-running the ANOVA with age as a between-subjects factor yielded only a main effect of subsequent memory [*F*(1, 42) = 69.65, *p* < 0.001, η_p_^2^ = 0.62], indicating that subjective ratings of conceptual combination success were diagnostic of subsequent memory in both age groups.

Another way to examine the relationship between EoD ratings and memory is to use conditional probabilities to compute the likelihood of a word pair being successfully recalled at test contingent upon it having been assigned a certain rating at study. As shown in Table 1, on average, older adults assigned one of the highest two ratings (5 and 6) to 50% of the word pairs. Of these word pairs, an average of 83% were successfully recognized during the memory test, versus 62% of the word pairs given an EoD rating of 4 or lower. Younger adults assigned an EoD rating of 5 or 6 to an average of 44%^1^ of the word pairs, and the probability of successful memory for these word pairs was 97% on average, versus 90% for word pairs given a rating of 4 or lower. A 2 × 2 ANOVA with factors Age (YA/OA) and pooled EoD rating (High: 5–6/Low: 1–4) on recognition probability yielded significant main effects of both Age [*F*(1, 46) = 29.00, *p* < 0.001, η_p_^2^ = 0.39] and EoD [*F*(1, 46) = 46.50, *p* < 0.001, η_p_^2^ = 0.50] as well as a significant interaction [*F*(1, 46) = 10.41, *p* = 0.002, η_p_^2^ = 0.19]. These analyses suggest that older adults’ memory performance dropped more precipitously than younger adults’ memory performance as subjective ease-of-definition decreased, which could reflect a steeper drop-off in combination difficulty itself and/or a greater difficulty encoding the more difficult-to-combine pairs into memory.

## Results: Electrophysiology

Effects of the task and W1 concreteness manipulations on ERPs were analyzed separately for first words (W1s) and second words (W2s) over the windows of 300–500 ms (N400) and 700–1000 ms (N700) respectively. Each analysis took the form of 2 (task: compare/combine) × 2 (concreteness: abstract/concrete) × 3 (electrode cluster: anterior frontal/frontocentral/parietal) ANOVAs. Effects involving electrode cluster are reported only in the context of interactions with other variables.

### Task and Concreteness Effects on First Words (W1s)

**FIGURE 4:**
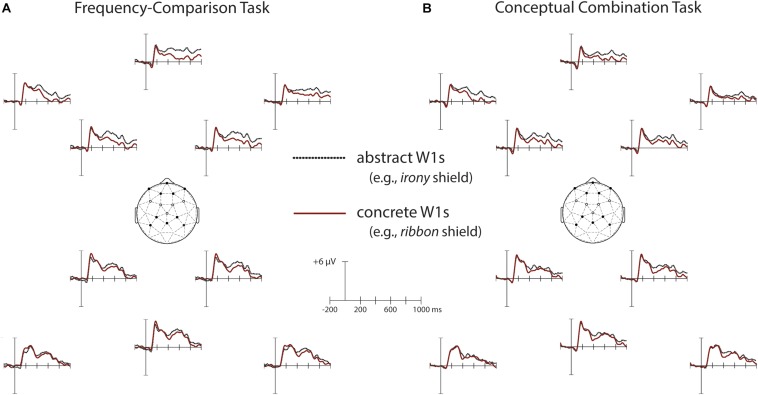
Grand average ERPs to concrete (e.g., “ribbon”) and abstract (e.g., “irony”) first words (W1s). Waveforms are shown from ten electrode sites, which comprise the anterior frontal and parietal clusters submitted to statistical analyses (see section Materials and Methods). Electrode positions are indicated on the head diagrams with solid black dots. The hollow black dots represent the frontocentral electrode cluster, which was also analyzed. **(A)** ERPs for W1s in the frequency-comparison task; **(B)** ERPs for W1s in the conceptual combination task. Significant N400 and N700 word-level concreteness effects were present in both tasks.

**FIGURE 5:**
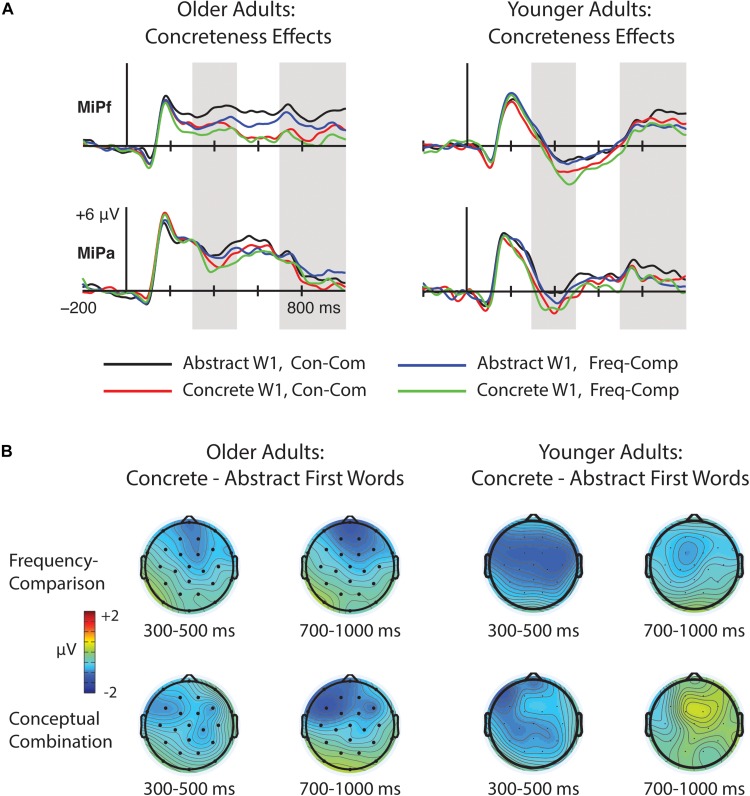
**(A)** Left: Grand average waveforms for abstract and concrete first words (W1s) in the frequency comparison and conceptual combination tasks at midline anterior frontal electrode MiPf and midline parietal electrode MiPa. Gray shading indicates the time windows used in N400 and N700 analyses. Right: Corresponding data from young adults reported in Lucas et al. (2017), plotted for comparison. **(B)** Topographical plots of concreteness effects over the time windows of interest in the frequency comparison and conceptual combination tasks in older adults (left), and in the younger adult sample for comparison (right).

We first compared N400 and N700 ERPs to concrete and abstract W1s in the frequency-comparison and conceptual combination tasks. The resulting waveforms are depicted in Figure 4, and relevant topographical plots can be found in Figure 5. The mean number of artifact-free trials per participant per condition was 33 (range = 22–36).

#### 300–500 ms

From 300 to 500 ms (N400), a significant main effect of W1 concreteness emerged [*F*(1, 23) = 10.15, *p* = 0.004, η_p_^2^ = 0.31], indicating more negative amplitudes for concrete than for abstract words. No other main effects or interactions were significant [*F*’s < 1.50, *p*’s >0.23]. These results suggest that concreteness effects for W1s were similar across the two tasks and broadly distributed over the head^2^.

#### 700–1000 ms

Analyses of ERPs over the 700–1000 ms (N700) window likewise revealed a significant main effect of W1 concreteness [*F*(1, 23) = 7.81, *p* = 0.01, η_p_^2^ = 0.25]. No other main effects or interactions approached significance, although a marginal Cluster × Task interaction [F(1.28, 29.44) = 3.19, *p* = 0.08, η_p_^2^ = 0.11] reflected a trend toward more negative ERPs in the combine relative to the compare condition that was larger at frontal relative to posterior electrodes.

#### Summary and Age Comparisons

In summary, older adults showed broadly-distributed concreteness effects for W1s that mirrored those previously found for young adults, in that ERPs were more negative for concrete than for abstract words from 300 to 500 ms regardless of task. In addition, concreteness effects in older adults persisted through the later time window of 700–1000 ms. To directly compare older adults’ concreteness effects with those reported for young adults in Lucas et al. (2017), we computed difference waves via a point-by-point subtraction of the ERP response to abstract W1s from the ERP responses to concrete W1s, collapsed across task and electrode cluster. Mean amplitudes of these differences for each time window were entered into a one-way ANOVA with age as a between-subjects factor. Age effects were non-significant for both windows [*F*(1, 46) = 0.22, *p* = 0.64, η_p_^2^ = 0.01 for 300–500 ms; *F*(1, 46) = 1.68, *p* = 0.20, η_p_^2^ = 0.04 for 700–1000 ms]. Thus, despite the appearance of larger N700 concreteness effects in the older relative to the younger adults, this age difference did not reach statistical significance.

In addition to the above mean amplitude-based comparisons, older and younger adults’ concreteness difference waves (ERP differences between concrete and abstract words) were entered into cluster mass permutation tests. Separate permutation tests were run on difference waves for the frequency comparison and conceptual combination tasks. All 10-ms time bins between 300 and 1000 ms and all fifteen electrodes were included. No significant differences were identified for either task (all cluster *p* > 0.41) Together, these analyses corroborate previous evidence that word-level concreteness effects are spared with age.

### Task and Concreteness-Modification Effects on Second Words (W2s)

**FIGURE 6:**
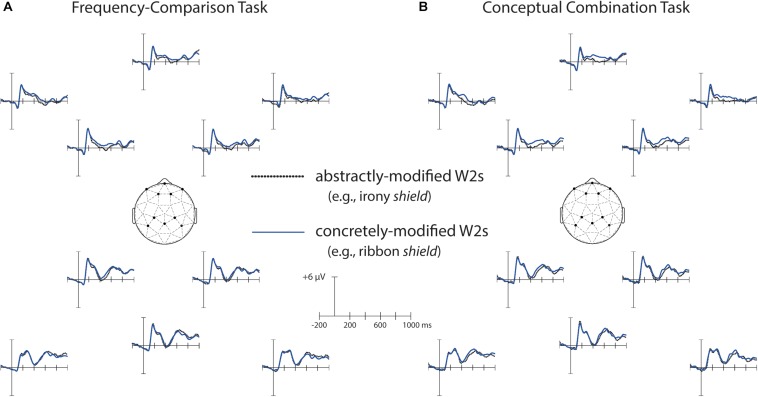
Grand average ERPs to concretely-modified and abstractly modified second words (W2s). Waveforms are shown from ten representative electrode sites whose positions are indicated on the head diagrams. **(A)** ERPs for W2s in the frequency-comparison task; **(B)** ERPs for W2s in the conceptual combination task. N400 compositional concreteness effects were present that did not vary by task. No compositional concreteness effects were present over the time window of the N700.

**FIGURE 7:**
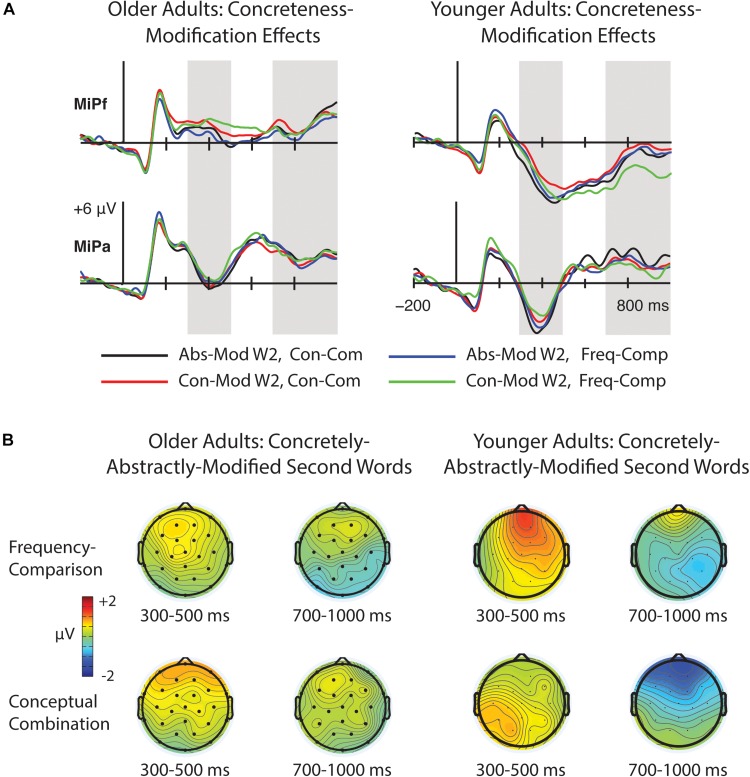
**(A)** Left: Grand average waveforms for abstractly-modified and concretely-modified second words (W2s) in the frequency comparison and conceptual combination tasks at midline anterior frontal electrode MiPf and midline parietal electrode MiPa. Gray shading indicates the time windows used in N400 and N700 analyses. Right: Corresponding data from young adults reported in Lucas et al. (2017), plotted for comparison. **(B)** Topographical plots of concreteness effects over the time windows of interest in the frequency comparison and conceptual combination tasks in older adults (left), and in the younger adult sample for comparison (right).

We next examined concreteness-modification effects present in both tasks by comparing ERPs to W2s that were preceded by concrete W1s with those that were preceded by abstract W1s. The resulting waveforms are depicted in Figure 6, and relevant topographical plots can be found in Figure 7. The mean number of artifact-free trials per participant was 32 for each condition (range = 19–36).

#### 300–500 ms

A significant main effect of W1 concreteness emerged from 300 to 500 ms [*F*(1, 23) = 7.60, *p* = 0.01, η_p_^2^ = 0.25], indicating more negative amplitudes for abstractly-modified than for concretely-modified words. Despite the appearance of larger concreteness-modification effects in the conceptual combination condition, the Task × W1 concreteness interaction was non-significant [*F*(1, 23) = 0.005, *p* = 0.95, η_p_^2^ < 0.01], nor was the three-way interaction [*F*(1.15, 26.39) = 0.74, *p* = 0.48, η_p_^2^ = 0.03]. No other main effects or interactions approached significance, although a marginal W1 concreteness × cluster interaction [*F*(1.32, 30.36) = 2.95, *p* = 0.08, η_p_^2^ = 0.11], revealed a trend toward greater effects of W1 concreteness at anterior relative to posterior electrode clusters.

#### 700–1000 ms

Analyses of ERPs to W2s from 700 to 1000 ms revealed no significant main effects or interactions for any variables in this time window (*F*’s < 1.65, *p*’s > 0.20).

#### Summary and Age Comparisons

In summary, compositional concreteness effects in older adults occurred in the N400 window, where they took the form of more negative ERPs to abstract versus concrete words. This pattern is similar to the N400 compositional concreteness effects observed in young adults in Lucas et al. (2017), and, indeed, a comparison of the mean difference in N400 amplitudes between concretely-modified and abstractly-modified W2s between age groups was non-significant [*F*(1, 46) = 0.12, *p* = 0.73]. Note that no main effects or interactions involving encoding task were significant in either age group, meaning that N400 compositional concreteness effects were present regardless of whether or not the participants were attempting conceptual combination. As such, these effects appear to be largely stimulus driven, and may result from the tendency of concrete W1s to activate a wider range of semantic features and thereby induce “happenstance” feature overlap with W2s to a greater extent than abstract W1s (see also Experiment 1 Discussion of Lucas et al., 2017).

Importantly, the older adults here showed no evidence of compositional concreteness effects in the N700 window. By contrast, younger adults in our previous study showed N700 compositional concreteness effects that were selective to the conceptual combination task, in which ERPs over the anterior frontal electrode cluster were more negative for concretely-modified relative to abstractly-modified W2s. Thus, we employed a one-way ANOVA to examine age differences in N700 compositional concreteness effects during this task over the anterior frontal cluster. The effect of Age was significant [*F*(1, 46) = 10.02, *p* = 0.003, η_p_^2^ = 0.10].

**FIGURE 8:**
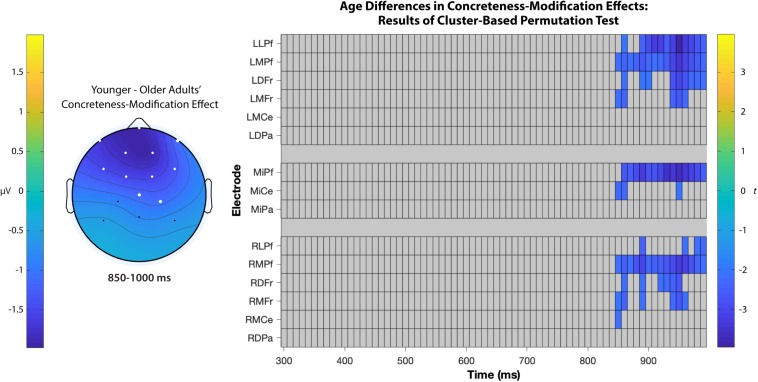
Left: Topographical plot of the difference between younger adults’ and older adults’ concreteness-modification effects (e.g., ERPs to W2s preceded by concrete versus abstract W1s) in the conceptual combination task from 850 to 1000 ms. White dots represent electrodes for which age differences emerged from the cluster-based permutation test. Right: Raster diagram representing the results of the permutation test, which included all displayed electrodes and all 10 ms intervals between 300 and 1000 ms. Electrode groupings along the y-axis correspond to left hemisphere (top group), midline (middle group) and right hemisphere electrodes (bottom group). Within each group, electrodes are listed in descending order of anteriority. Blue rectangles indicate electrodes and time points in which young adults’ concreteness-modification effects are significantly more negative than those of older adults.

As with the word-level concreteness effects, we also examined age differences in compositional concreteness effects using cluster-based permutation analyses over all electrodes and all 10 ms time bins from 300 to 1000 ms separately for the frequency comparison and conceptual combination task. No difference was identified in the frequency comparison task (*p* > 0.51). However, the permutation test for the conceptual combination task identified a significant difference between age groups (*p* = 0.03) in the form of a negative frontally-focused cluster, which began around 850 ms and continued to the end of the epoch (see Figure 8). Note that cluster-based permutation analyses control the type 1 error rate only with respect to whether or not the overall multivariate datasets differ (i.e., the null hypothesis is that the compositional concreteness effects of the older and younger adults are “interchangeable”), rather than for each individual time point/electrode pairing. Thus, these results should be interpreted as an approximate rather than exact spatiotemporal locus of the age difference (Sassenhagen and Draschkow, 2019). Nonetheless, the overall timing and topography of this cluster is consistent with the N700 effect found in the time window-based age comparison.

### Single-Trial Analyses

Like the young adults in Lucas et al. (2017), older adults here showed improvements in associative recognition memory in the conceptual combination blocks relative to the frequency-comparison blocks. However, when comparing the recognition performance of the two age groups, it is apparent that conceptual combination did not reduce age-related memory deficits relative to the frequency-comparison task. Rather, age differences were numerically larger for concrete-concrete word pairs in the combination versus the frequency task, and were significantly larger for abstract-concrete word pairs, suggesting a reduction in the effectiveness of conceptual combination as a memorization strategy. Likewise, as in Huang et al. (2012), older adults did not show compositional concreteness effects on N700 ERPs, suggesting a reduced tendency or ability to use features of the W1 to modify the processing applied to the W2.

A clear next question concerns the nature of the relationship of the neurocognitive processing reflected in N700 potentials to conceptual combination and associative memory formation. One possibility is that N700 amplitudes track the relative success of conceptual combination and memory formation from trial to trial, such that amplitudes are more negative for trials on which conceptual combination is easiest. This pattern, together with the findings that word pairs with more imageable W1s were both rated as easier to combine and were better remembered, would seem intuitive in light of imagery-based accounts of N700 concreteness effects.

That said, other recent findings complicate the notion that the ease or vividness of the evoked imagery *per se* is the primary determinant of N700 concreteness effects. The “canonical” effect (more negative N700 amplitudes to concrete words) has typically been found in studies that ask participants to judge whether or not a word is easily imageable (West and Holcomb, 2000; Gullick et al., 2013) or in tasks that require semantic processing without explicit reference to imagery (Holcomb et al., 1999; Huang et al., 2010). However, Welcome et al. (2011) obtained a reversal of this pattern when participants were asked to create an image for each and every word before moving on to the next, regardless of difficulty. In this context, N700 amplitudes were greater for abstract word pairs, suggesting a sensitivity to the amount of effort or cognitive resources put toward image generation on a given trial. In addition, Barber et al. (2013) reported evidence that N700 concreteness effects may not exclusively index processes related to visual imagery, but may reflect a more general top-down process of retrieving and integrating intrinsic features (visual and otherwise) that tends to be engaged to a greater extent during the processing of feature-rich concrete words. Together, these findings raise the possibility that more difficult to combine word pairs may evoke *larger* N700 amplitudes, at least in young adults, insofar as they trigger additional cognitive control processes to overcome the difficulty.

To gain traction on this issue, we used linear mixed-effects modeling to model W2 N700 amplitudes at the individual trial level based on participants’ ease-of-definition (EoD) ratings during the conceptual combination task. Amplitudes for each trial reflect the mean voltage from 700–1000 ms, averaged over all electrodes in the anterior frontal cluster. Analyses were carried out using the lme4 software package version 1.1-19 (Bates et al., 2015) and the afex package version 0.23-0 (Singmann et al., 2015). We first constructed a statistical model that included: (1) age and W1 concreteness as categorical fixed-effect predictors, (2) EoD rating as a continuous fixed-effect predictor, with higher ratings indicating lower perceived difficulty, (3) interactions among fixed effects, and (4) a random intercept for participants. Random slopes were removed to facilitate model convergence. Prior to analyses, the continuous predictor was mean-centered within each age group and the nominal predictors were sum coded. Effect significance was evaluated using Satterthwaite’s approximation as implemented in the lmerTest package (Kuznetsova et al., 2017).

The results are depicted in Table 2. As shown, W1 concreteness was a significant predictor of N700 amplitudes (β = −0.29, *t* = 1.99, *p* = 0.047), with concrete items eliciting larger N700 amplitudes than abstract items. Consistent with the trial-aggregated analyses, this effect was qualified by a significant age × W1 concreteness interaction (β = 0.35, *t* = 2.40, *p* = 0.02). The main effect of EoD rating was also significant (β = 0.23, *t* = 2.70, *p* = 0.007) and also interacted with age (β = −0.18, *t* = 2.08, *p* = 0.04). Note that the positive parameter estimate for the main effect indicates that N700 amplitudes were smaller (less negative) for trials that received higher ease-of-definition ratings. The EoD rating × W1 concreteness interaction was not significant (β = −0.09, *t* = −1.11, *p* = 0.27), nor was the three-way interaction (β = 0.01, *t* = 0.15, *p* = 0.88).

**TABLE 2:** Results of linear mixed-effects models predicting W2 N700 amplitudes during the conceptual combination task.

**Parameter**	**Estimate**	**SE**	***t*-value**	***P-*value**
Intercept	–0.36	0.43	0.83	0.41
Age	1.16	0.43	2.70	0.01^∗∗^
W1 concreteness	–0.29	0.15	1.99	0.047^∗^
EoD rating	0.23	0.09	2.71	0.007^∗∗^
Age × W1 concreteness	0.35	0.15	2.40	0.02^∗^
Age × EoD rating	–0.18	0.09	2.08	0.04^∗^
W1 concreteness × EoD rating	–0.09	0.08	1.11	0.27
Age × W1 concreteness × EoD	0.01	0.08	0.15	0.88

**Parameter estimates are for fixed effects involving age, W1 concreteness, and Ease-of-Definition (EoD) rating. T- and p-values obtained using Satterthwaite’s approximation. SE, standard error; ^∗^p < 0.05; ^∗∗^p < 0.01.**

**FIGURE 9:**
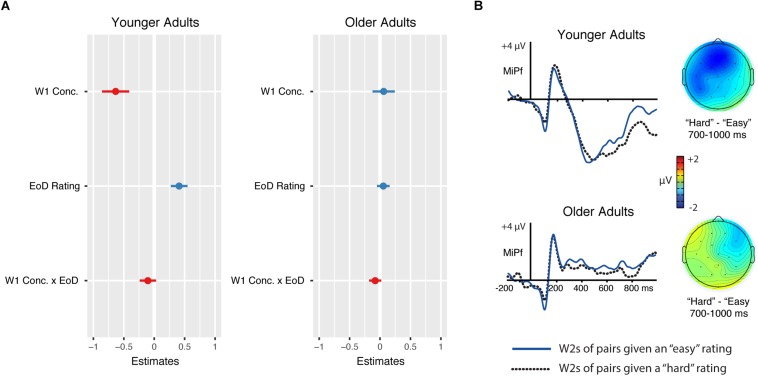
**(A)** Fixed-effect parameter estimates for the effects of W1 concreteness (W1 Conc.), Ease-of-Definition rating (EoD Rating), and the interaction term (W1 Conc. × EoD) for the younger adults (left) and the older adults (right) on W2 N700 amplitudes in the conceptual combination task. The dots correspond to the parameter estimates, and the lines denote standard errors. The two significant main effects in the younger adults reflect greater negativities for: (1) concretely-modified relative to abstractly-modified W2s, and (2) W2s for word pairs rated as harder versus easier to define (e.g., given lower EoD ratings, using 6-point rating scale as a continuous predictor). **(B)** For visualization purposes only, ERPs to W2s are plotted at midline anterior frontal electrode MiPf for pairs rated as “hard” (given a rating of four or lower on the ease-of-definition scale) and for pairs rated as “easy” (given an EoD rating of five or six). The 1–4 and 5–6 cut-offs were chosen because participants in both age groups assigned a “5” or “6” to approximately half of the trials. Topographical plots reflect the difference between “hard” and “easy” trials over N700 window.

To further examine the effects involving EoD rating, we tested separate models for each age group that included W1 concreteness and EoD rating as fixed effects and a random intercept for participant. Figure 9A depicts the parameter estimates and standard errors of the resulting models. As shown, both main effects were significant for the younger adults (β = −0.64, *t* = 2.85, *p* = 0.005 for W1 concreteness; β = 0.41, *t* = 2.99, *p* = 0.003 for EoD rating), whereas the interaction was non-significant (β = −0.11, *t* = 0.77, *p* = 0.44). These results indicate that younger adults’ W2 N700 amplitudes were: (1) more negative for concrete-concrete than abstract-concrete trials, regardless of difficulty rating, and (2) more negative for trials that received more difficult ratings (e.g., lower EoD ratings), regardless of W1 concreteness. The analogous model in the older adults revealed no significant main effects, nor a significant interaction (all *p*’s > 0.40). For visualization purposes only, Figure 9B depicts W2 waveforms from the conceptual combination task in either age group subdivided by subjective rating (based on a split in which ratings 1:4 were grouped as “hard” and 5:6 as “easy,” see figure caption).

## Discussion

Adult aging brings about changes to multiple domains of cognition, including aspects of memory and language processing. In the domain of memory, there is evidence that relational or associative memory is more vulnerable to the negative effects of age than is memory for single items (e.g., Old and Naveh-Benjamin, 2008). An analogous distinction is present in the literature on language and aging: while older and younger adults generally show similar lexical-level processing, aging has been linked to changes in compositional language processing (Wlotko et al., 2010). Thus far, however, these two facets of neurocognitive aging have been studied largely independently of one another. Indeed, despite decades of research on the importance of “deep” or meaning-based processing for memory, little attention has been paid to potential ways in which reduced online semantic integration may affect associative memory performance in older adults.

The present study addresses a question that falls squarely at the memory-language interface: the extent to which older and younger adults differ in implementing an associative memorization strategy that relies on conceptual combination. Previous work (Ahmad et al., 2014) has found that age-related associative memory deficits are smaller for noun pairs that corresponded to pre-experimentally familiar compound words (e.g., “store keeper”) relative to those that do not, presumably because familiar pairs are represented in memory in a “unitized” or item-like manner. However, initial studies examining older adults’ ability to engage in *ad hoc* unitization of non-pre-experimentally related stimuli have yielded mixed results (c.f. Bastin et al., 2013; Kamp et al., 2018). For example, a recent examination of older adults’ ability to use experimenter-provided definitions to aid in the memorization of novel word pairs (Kamp et al., 2018) found that age differences in performance on subsequent associative memory tests were exaggerated rather than reduced relative to a control condition^3^. There is thus a need to better understand how aging intersects with the neurocognitive demands of flexible language processing in order to determine whether and when this and other language-mediated strategies are appropriate.

In the present study, we adapted a paradigm previously used to study the processing of adjective-noun modification relationships (Huang et al., 2010, 2012) to examine noun-noun combination for unrelated word pairs. A key feature of this design is the systematic manipulation of the concreteness of the first noun in each pair, which has been shown in young adults to induce compositional concreteness effects on a slow frontal potential termed the N700 (Lucas et al., 2017). Notably, in the present study, N700 compositional concreteness effects were absent in older adults, despite the fact that word-level concreteness effects were age-invariant. Moreover, while older adults’ associative memory was superior following the conceptual combination task relative to a non-combinatory encoding task, the magnitude of the age-related deficit was either similar between the two tasks (for concrete-concrete pairs) or greater in the conceptual combination task (for abstract-concrete pairs). As such, these data join others to suggest that there may be limits to older adults’ ability to use conceptual combination as an encoding strategy.

As previously discussed, N700 amplitudes have been related in previous work to the retrieval and integration of semantic features – including, but perhaps not limited to, features involved in visual imagery (West and Holcomb, 2000; Welcome et al., 2011; Barber et al., 2013; Gullick et al., 2013). It therefore seems plausible that older adults may either be less able or less inclined to use compositional imagery or other semantic integration processes to aid in conceptual combination. Indeed, our single-trial analyses provide evidence that W2 N700 potentials are sensitive to variability in perceived difficulty of conceptual combination. In young adults, W1 concreteness and perceived difficulty accounted for independent variance in W2 N700 amplitudes: amplitudes were larger (more negative) for concrete-concrete word pairs regardless of rated difficulty and for word pairs that receive greater difficulty ratings regardless of W1 concreteness. This pattern of results coheres with proposals that attribute N700 concreteness effects to the greater number of features intrinsic to concrete relative to abstract concepts (e.g., Wiemer-Hastings and Xu, 2005), which places greater demands on certain semantic integration processes. By this interpretation, the augmented N700 amplitudes to the W2s for more difficult word pairs (in the young adults) could reflect the engagement of additional processing resources to aid conceptual combination. Likewise, the absence of W2 N700 modulation by either variable in older adults is consistent with an overall impoverishment of semantic integration at the compositional level.

An important direction for future research will be to more directly examine how variability in N700 amplitudes relates to subsequent associative memory at the trial level. While both age groups were significantly more likely to remember word pairs that were given higher versus lower EoD ratings, recognition accuracy in the younger adults was quite high across the board. Accordingly, it is possible that the N700 enhancement in younger adults for more difficult pairs reflected a process that helped to “rescue” memory formation on these trials. This possibility could be tested in future work that compares encoding-related ERPs for later-remembered and later-forgotten trials in young adults separately for trials rated as easy versus difficult to combine, with the prediction that N700 potentials would selectively predict memory for difficult trials.

Interestingly, older adults’ subjective ratings and associative memory performance suggest disproportionate difficulty applying the conceptual combination strategy to the abstract-concrete word pairs. This is an unusual finding: although it is well-established that concrete words enjoy facilitated processing speed and memory relative to abstract words, older adults have generally shown concreteness effects that are either smaller than or equal to those of younger adults (e.g., Whitbourne and Slevin, 1978; Rissenberg and Glanzer, 1987; Dirkx and Craik, 1992; Peters and Daum, 2008; Roxbury et al., 2015). As such, further research will be necessary to identify the neurocognitive locus of older adults’ increased difficulty creating combinations using abstract modifiers.

Cognitive theories of conceptual combination – such as the Competition Among Relations in Nominals (Gagné and Shoben, 1997) and Relational Interpretation Competitive Evaluation theories (Gagné and Spalding, 2013) – point to differences in the “combinatorial histories” of modifiers as a factor that may influence their amenability to flexible conceptual combination. According to these models, when a modifier is presented as part of a compound, multiple possible relations are activated based on that word’s history as a modifier, and competition among these relations must be resolved before a compound can be perceived as meaningful (see Schmidtke et al., 2016, for supporting evidence from two lexical decision megastudies). It seems plausible that the process of resolving relational competition may pose more difficulty for older adults, perhaps due to a combination of greater word knowledge (e.g., more competing meanings activated) and the need to engage in control processes to choose among competing meanings. As such, avenues for future research may include examinations of: (1) whether abstract and concrete modifiers differ in the number and/or availability of “suggested” possible relations, and (2) whether older adults are disproportionately hindered by increases in competition among possible relations.

An additional consideration pertains to the *type* of relations suggested by abstract and concrete modifiers. Recent neuroimaging evidence (Boylan et al., 2017) suggests that partially distinct brain networks are involved in feature-based or attributive interpretations of the conceptual combinations (e.g., “monster truck,” which denotes a truck with monster-like features) relative to relationship-based interpretations (e.g., “soup spoon,” which denotes a modifier-head relationship that is not based on shared features). It is possible that abstract modifiers have a greater tendency to evoke relationship-based interpretations relative to more feature-rich concrete modifiers, and that it is this distinction that accounts for the age differences observed here. Future research could also examine this possibility, although at least one study (Taler et al., 2005) provides behavioral evidence against the idea that aging selectively impacts relationship-based interpretations of novel compounds.

Another goal for future research is to test the generality of these findings by juxtaposing conceptual combination with a wider range of alternative encoding strategies. We chose the frequency rating task as our starting point because it requires participants to engage in “deep” or meaning-based processing of both items in each pair without encouraging these meanings to be combined. The inclusion in future studies of strategies that vary in their neurocognitive overlap with conceptual combination will provide more precise insight into the underpinnings of the observed age differences, both in encoding-related ERPs and associative memory performance^4^. For example, it may be informative to compare the conceptual combination strategy with interactive imagery (e.g., imagining the two items interacting in some way), which arguably requires the use of compositional imagery without requiring the generation and selection of relational interpretations.

Multiple existing theories of age-related memory deficits point to a reduction in the use of strategies as a contributing factor to worsening associative memory (e.g., Glisky et al., 2001; Luo et al., 2007; Naveh-Benjamin et al., 2007; Craik and Rose, 2012; Hertzog et al., 2012). In particular, the Associative Deficit Hypothesis (Naveh-Benjamin, 2000; Naveh-Benjamin et al., 2007) implicates a reduced tendency of older adults to initiate intentional strategies (such as sentence-generation or interactive imagery) that aid in the formation of new associative memories. An area of debate within this literature concerns the extent to which these deficits are specific to the spontaneous initiation of strategies, rather than the ability to implement them when given explicit instructions to do so. In the present study, older adults were not only provided with strategy instructions, but were also offered a brief training and practice in both the conceptual combination and frequency comparison strategies. As such, our results provide evidence that: (1) strategy-based deficits with age are not limited to spontaneous initiation, and (2) certain combinations of strategies and stimulus properties (e.g., conceptual combination for abstract-concrete word pairs) may present particular difficulties for older adults. More generally, this work underscores the idea that age effects on compositional processing in language and associative memory are not independent of one another and highlights a need for further research on interactivity between these cognitive domains across the lifespan.

## Data Availability Statement

The datasets generated for this study are available on request to the corresponding author.

## Ethics Statement

The studies involving human participants were reviewed and approved by the Institutional Review Board at the University of Illinois Urbana-Champaign. The patients/participants provided their written informed consent to participate in this study.

## Author Contributions

HL, RG, RH, and KF contributed to the conception and design of the study, and wrote the manuscript. HL and RG collected the data. HL performed the statistical analysis. All authors contributed to the manuscript revision, and read and approved the submitted version.

## Conflict of Interest

The authors declare that the research was conducted in the absence of any commercial or financial relationships that could be construed as a potential conflict of interest.

## Footnotes

1 The proportion of trials given a 5 or 6 rating did not differ significantly by age [*t*(39.97) = 1.27, *p* = 0.21, Cohen’s *d* = 0.37]. Of course, one cannot assume that the “absolute” difficulty level of word pairs assigned a given rating is equal between age groups, particularly since all participants were encouraged to use the entire rating scale.

2 Note that the sustained frontal concreteness effect captured in the N700 window appears to begin somewhat earlier, overlapping with the N400 window. Thus, it is possible that both components are contributing to the concreteness effects measured over this window.

3 Age differences were also found in ERPs recorded during encoding in the study by Kamp et al. (2018). However, comparisons with the present ERP effects are complicated by significant methodological differences between studies.

4 Another option is to include a “no strategy” condition in which participants are given no instructions other than to attempt to remember the word pairs. However, previous research suggests that the nature and extent of spontaneous strategy use can differ considerably between younger and older adults under these circumstances (e.g., Dunlosky and Hertzog, 2001; Naveh-Benjamin et al., 2007; Tournier and Postal, 2011; Craik and Rose, 2012). Thus, this type of design may be more suitable to answer questions about age effects on self-initiated strategy use.
